# Macrophages in T cell-mediated rejection of renal allografts: pathological mechanisms and CAR-macrophage immunotherapy

**DOI:** 10.1080/0886022X.2026.2753269

**Published:** 2026-11-04

**Authors:** Guohang He, Jianbo Qing, Guming Chen, Shurui Wang, Zixuan Qu, Xiuyan Yang, Hongfeng Huang, Haifeng Gu, Karim Soliman, Hong Jiang

**Affiliations:** ^a^ Kidney Disease Center, the First Affiliated Hospital, College of Medicine, Zhejiang University, Hangzhou, Zhejiang, P.R. China; ^b^ Institute of Nephropathy, Zhejiang University, Hangzhou, Zhejiang, P.R. China; ^c^ Zhejiang Clinical Research Center of Kidney and Urinary System Disease, Hangzhou, Zhejiang, P.R. China; ^d^ Cheeloo College of Medicine, Shandong University, Jinan, Shandong, P.R. China; ^e^ Department of Nephrology, Sir Run Run Shaw Hospital, Zhejiang University School of Medicine, Hangzhou, Zhejiang, P.R. China; ^f^ Department of Geriatrics, The First Affiliated Hospital, Zhejiang University School of Medicine, Hangzhou, Zhejiang, P.R. China; ^g^ XiangYa School of Medicine, Central South University, Changsha, Hunan, P.R. China; ^h^ Department of The Second Clinical School of Medicine, Anhui Medical University, Hefei, Anhui, China; ^i^ Thomas E. Starzl Transplantation Institute/UPMC, University of Pittsburgh, Pittsburgh, PA, USA

**Keywords:** Renal transplantation, T cell-mediated rejection, macrophages, immune microenvironment, macrophage-to-myofibroblast transition, chimeric antigen receptor macrophages

## Abstract

Acute T cell-mediated rejection (TCMR) remains an important cause of early and intermediate-term graft dysfunction after kidney transplantation. As effector cells that bridge innate and adaptive immunity, macrophages are deeply implicated in the initiation, progression, and fibrotic sequelae of TCMR. This review synthesizes their multifaceted pathological roles: antigen presentation and T cell activation during early rejection, pro-inflammatory polarization driving parenchymal injury, chemokine-mediated recruitment that sustains immune infiltration, and macrophage-to-myofibroblast transition (MMT) underlying interstitial fibrosis in chronic graft deterioration. Because conventional immunosuppression is systemic and incompletely addresses the intragraft immune microenvironment, we further examine chimeric antigen receptor macrophages (CAR-Ms) as an emerging strategy for TCMR intervention. Building on current mechanistic insight, we propose two engineering approaches: targeted-elimination CAR-Ms designed to recognize and clear pathogenic effector immune cells, and tolerance-inducing resident CAR-Ms engineered to suppress inflammation and fibrosis while promoting tolerance. Together, these macrophage-engineering strategies provide a conceptual approach to reshaping the renal allograft immune microenvironment and improving long-term graft survival.

## 1. Introduction

### 1.1. Renal allotransplantation and the challenge of acute rejection

Kidney transplantation is the preferred treatment for end-stage renal disease (ESRD), restoring renal function and markedly improving long-term survival and quality of life. Donor-recipient genetic disparity exposes the graft to alloimmune recognition and, in some recipients, rejection [1].

Rejection follows distinct temporal patterns and is classified into four types based on onset, clinical course, and underlying mechanism: hyperacute, accelerated, acute, and chronic [2]. Acute rejection (AR) typically arises within the 1 week to 6-month post-transplant, though widespread use of triple immunosuppression, calcineurin inhibitors such as tacrolimus combined with antimetabolites such as mycophenolic acid, has progressively delayed its onset.

Acute rejection reflects recipient immune recognition of donor alloantigens [3]. Recipient immune cells identify donor MHC/HLA molecules on the graft as foreign, triggering a coordinated response driven chiefly by adaptive immunity but with substantial innate contribution [4]. As a principal determinant of early and intermediate-term graft dysfunction and loss, the mechanisms, diagnosis, and management of acute rejection remain a focus of transplant immunology [5].

### 1.2. Pathological progression and clinical consequences of acute T cell-mediated rejection (TCMR)

Based on the effector mechanisms and tissues involved, acute renal allograft rejection is classified into four phenotypes: T cell-mediated rejection (TCMR), antibody-mediated rejection (ABMR), mixed rejection, and subclinical rejection (SCR) [6].

ABMR arises from *de novo* donor-specific antibodies (DSAs) that target graft vascular endothelium and activate complement, producing microvascular inflammation and endothelial injury [7]. TCMR, by contrast, results from direct graft injury mediated by recipient adaptive and innate immune cells rather than circulating antibodies [8]. It is marked by extensive immune infiltration and tissue destruction, with T cells as the dominant effector population [9]. Mixed rejection denotes the coexistence of TCMR and ABMR, with simultaneous cellular and humoral activation, while SCR describes histological evidence of TCMR or ABMR on protocol biopsy in the absence of overt functional decline [10,11]. Because macrophages participate in both cellular and humoral components of allograft injury, macrophage-directed therapeutic strategies may also have potential relevance to mixed rejection rather than being restricted to isolated TCMR. In this setting, modulation of macrophage function could theoretically complement therapies targeting antibody-mediated injury, although this possibility remains to be validated experimentally.

Although ABMR has received increasing attention, TCMR remains clinically and mechanistically important: It is both a trigger for subsequent humoral activation and antibody production, and a process mediated by complex immune-cell network that may be reversible when recognized and treated promptly. TCMR therefore continues to anchor efforts to define rejection mechanisms and develop new therapies.

Histologically, TCMR produces extensive cortical interstitial edema, tubular injury, and rarefaction of peritubular capillaries, culminating in irreversible parenchymal atrophy and cortical fibrosis [12]. These structural changes impair filtration capacity and accelerate chronic graft dysfunction, shortening graft survival [13]. Graft failure can return patients to ESRD, with attendant electrolyte disturbances, severe anemia, and cardiovascular complications that substantially erode overall health [14]. The resulting burden of hospitalization, dialysis reinitiation, and repeat allocation also imposes considerable strain on healthcare systems [6].

This progressive structural and functional decline is driven by a coordinated assault from multiple interconnected recipient immune populations [15]. Among them, macrophages, effector cells with dual roles in innate defense and adaptive immune regulation, are indispensable to allograft rejection [16]. TCMR pathogenesis is thus not solely a matter of antigen-specific lymphocyte responses; macrophages substantially shape antigen presentation, inflammatory polarization, tissue injury, and subsequent fibrosis at every stage [17].

This review expands the conventional T cell-centric view of TCMR to examine the mechanistic diversity and functional heterogeneity of macrophages in acute cellular rejection, to provide a mechanistic framework for macrophage-directed immunomodulatory strategies in renal transplantation.

## 2. Developmental origins and functional heterogeneity of macrophages in the renal allograft microenvironment

Macrophages infiltrating the renal allograft during the onset and progression of TCMR are heterogenous. Rather, they comprise distinct subsets differing in developmental origin, phenotype, and function, all of which shift dynamically in response to local immune cues.

Developmentally, two major sources supply macrophages to the transplanted kidney. Donor-derived tissue-resident macrophages (TRMs), seeded from embryonic yolk sac or fetal liver progenitors, persist as long-lived populations within the cortical and medullary interstitium under steady-state conditions. Marked by CX3CR1 and CD81 expression, these cells normally maintain tissue homeostasis and support microvascular endothelial transport; after transplantation, they additionally act as early sentinels that sense and capture alloantigens within the graft.

Recipient-derived monocyte-derived macrophages (MDMs), by contrast, arise from bone marrow CD14^+^CD16^-^ classical monocytes recruited during ischemia-reperfusion injury or alloimmune inflammation. Guided by chemokine gradients, most notably the CCL2/MCP-1 axis, these monocytes extravasate into the renal interstitium and rapidly differentiate into effector macrophages. They come to dominate the intragraft macrophage pool during rejection and serve as the principal drivers of local inflammatory amplification and tissue damage [18].

Macrophages were traditionally divided into classically activated, inflammatory M1 cells, and alternatively activated M2 cells associated with repair but also potentially with fibrosis [19]. Single-cell RNA sequencing and spatial transcriptomics have since overturned this binary view, showing a continuum of transcriptional states and multiple subsets with distinct molecular signatures and specialized functions within the transplant microenvironment [20].

Accordingly, in this review, the M1/M2 terminology is used as a functional and approximate descriptor rather than as a definition of discrete or stable macrophage identities. When discussing studies that originally adopted this classification framework, we retain the M1/M2 nomenclature to preserve fidelity to the experimental literature. Specifically, macrophages exhibiting these functional features are referred to as ‘M1-like’ or ‘M2-like’ macrophages, whereas the directional processes by which macrophages acquire such features are described using the conventional terms ‘M1 polarization’ and ‘M2 polarization’.

Renal allograft macrophages are therefore best understood as a dynamic, heterogenous cellular compartment, combining donor-resident and recipient-recruited cells, whose phenotypes are continuously reshaped by local environmental signals. Different subsets engage at different stages of rejection, from alloantigen recognition through inflammatory amplification, parenchymal injury, and tissue remodeling. The spatial and temporal trajectory of these macrophage states may thus be a determinant of TCMR onset, progression, and clinical outcome [21].

## 3. Molecular mechanisms underlying macrophage-mediated regulation of TCMR progression

Macrophages in TCMR are not merely terminal effectors of tissue injury; their phenotype and function evolve continuously across immune activation, inflammatory amplification, and dysregulated repair [22]. Their pathological contributions span several interconnected processes: driving alloantigen presentation and T cell activation, sustaining pro-inflammatory polarization, perpetuating immune cell recruitment, and, in the later phase of rejection, governing tissue remodeling and fibrosis [23].

### 3.1. Antigen presentation and T cell activation

During the initiation phase of rejection, graft-infiltrating macrophages act as professional antigen-presenting cells (APCs). Expressing MHC class II and costimulatory molecules such as CD80 and CD86, they capture and present donor alloantigens, supplying the signals required to activate and expand antigen-specific CD4^+^ and CD8^+^ T cells.

This antigen-presenting function is shaped by several receptor- and pathway-level checkpoints. As recipient monocytes infiltrate the graft, CD47/SIRPα signaling within the allogeneic microenvironment markedly induces leukocyte immunoglobulin-like receptor B2 (LILRB2), which then engages donor HLA class I molecules to upregulate MHC-II expression and boost antigen-presenting capacity [24]. In parallel, ischemia-reperfusion injury releases damage-associated molecular patterns (DAMPs) that sustain the myeloid TLR4/HIF-1α axis, a second pathway maintaining the expansion and antigen-presenting activity of MHC-II^+^ pro-inflammatory macrophages [25].

Countervailing inhibitory and senescence-linked mechanisms temper this activation. After cold ischemia and reperfusion injury, donor-resident macrophages sharply upregulate the inhibitory receptor IL-1R8, which dampens TLR4-driven activation, curbs antigen presentation and T cell priming, and favors an M2-like shift [26]. In older recipients, accumulating p21^high^ senescent macrophages express elevated Zfp36, an RNA-binding protein that binds the 3′ UTR of IL-27 p28 mRNA and accelerates its degradation. Because IL-27 promotes CD8^+^ T cell activation, this loss blunts CD8^+^ T cell proliferation and cytotoxicity, which may contribute to the lower incidence of acute cellular rejection observed in elderly recipients (Figure 1) [27].

**Figure 1.**
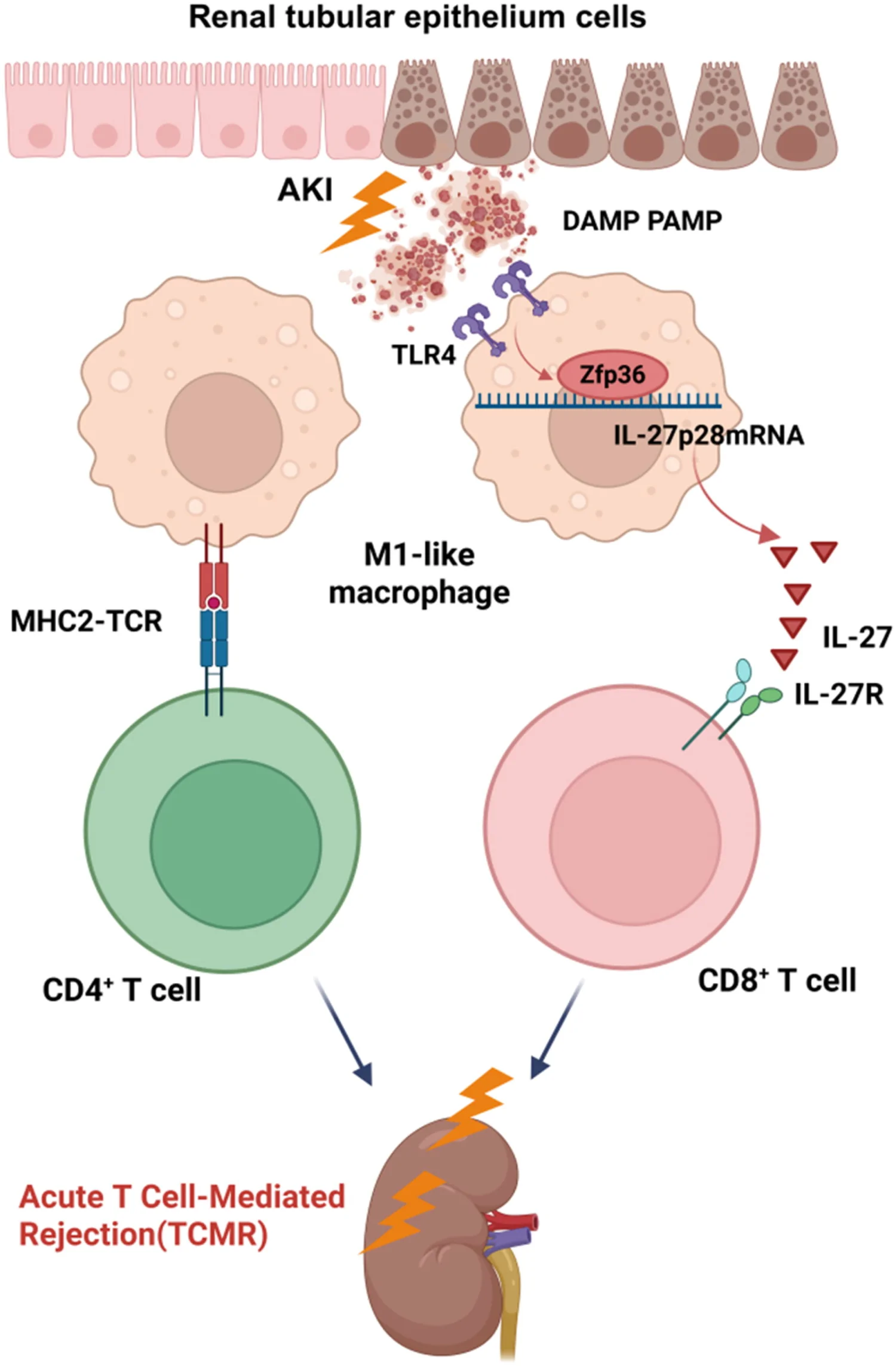
**Macrophage-Mediated Antigen Presentation and T-Cell Activation in Renal Allograft TCMR.** Renal tubular injury releases DAMPs/PAMPs that activate macrophages through TLR4 signaling. Macrophages promote CD4**^+^** T-cell activation through MHC-II–TCR interactions, while the Zfp36/IL-27 axis modulates CD8**^+^** T-cell responses, collectively influencing acute TCMR.

Together, the LILRB2, TLR4/HIF-1α, and IL-1R8 pathways coordinately set the magnitude of early T cell responses in TCMR and identify candidate pathways for modulating early alloimmune activation.

### 3.2. M1 polarization and macrophage-mediated renal parenchymal injury

Once T cells are activated, the resulting inflammatory milieu promotes a pro-inflammatory phenotype: IFN-γ and TNF-α released by activated T cells drive classical M1 polarization. M1-like macrophages amplify graft inflammation through abundant cytokine production, worsening cortical interstitial inflammation and tubulitis, while their release of reactive oxygen species and nitric oxide directly damages tubular epithelial and peritubular capillary endothelial cells, contributing to acute tubular injury and necrosis [28].

This inflammatory program involves transcriptional regulation, metabolic reprogramming, and inflammasome activation. In the inflammatory milieu, ERK1/2-FOS signaling upregulates phosphoglycerate dehydrogenase (PHGDH), which sustains TLR4 expression and activates the NLRP3/caspase-1 inflammasome, driving caspase-1 cleavage and the release of mature IL-1β, IL-18, and macrophage-derived IL-23a, mediators that further reinforce M1 polarization. IL-23a in particular promotes CD8^+^ T cell activation and granzyme B production, amplifying cytotoxic T cell responses and worsening tubular epithelial death and tubulitis [29]. Concurrently, DAMPs released during ischemic injury sustain macrophage TLR4 signaling and downstream HIF-1α-dependent transcription, further favoring M1 differentiation [25].

Injured tubular epithelial cells mount a countervailing stress response. Activation of the transcription factor ATF4 induces paracrine secretion of GDF15, which acts on infiltrating macrophages *via* Smad2/3 signaling to suppress M1 polarization and promote an anti-inflammatory, reparative M2-like functional phenotype, dampening inflammatory cascades, limiting further infiltration, and limiting tubular injury [30]. Endogenous metabolites contribute similarly: D-kynurenine (D-Kyn), more immunosuppressive than its L-enantiomer, inhibits the ERK1/2-FOS-PHGDH axis, thereby suppressing NLRP3 inflammasome activation, reducing IL-23a production, and limiting CD8^+^ T cell-mediated injury [29].

The pro-inflammatory PHGDH–NLRP3 axis and the protective, GDF15-mediated counter-program thus operate in dynamic opposition, and the balance between them may influence the extent of renal allograft injury and the preservation of tissue homeostasis during TCMR.

### 3.3. Chemokine-mediated amplification and sustained immune cell recruitment

As rejection progresses, the local immune response can evolve from initial cellular activation into sustained recruitment and amplification. Macrophages upregulate the C-X-C chemokines CXCL9 and CXCL10, which engage CXCR3 on activated effector T cells to establish a positive feedback loop of reciprocal macrophage–T cell recruitment, driving persistent immune cell accumulation within the cortical interstitium.

Single-cell RNA sequencing and spatial transcriptomics indicate that this recruitment network is largely orchestrated by a specialized macrophage subset. Within rejecting allografts, CD47/SIRPα signaling in the inflammatory microenvironment induces LILRB2 expression in infiltrating monocytes, steering a subset of recipient-derived MDMs toward a CXCL10^+^LILRB2^+^ pro-inflammatory phenotype. By recognizing donor HLA class I molecules, macrophage LILRB2 sustains effector T cell expansion and alloimmune inflammation, worsening tubular injury, microvascular damage, and interstitial inflammation. This CXCL10^+^LILRB2^+^ population is a major source of rejection-associated mediators, including CXCL9, CXCL10, WARS, and GBP1, and its abundance correlates independently with rejection severity and long-term graft dysfunction risk, underscoring its role in recruiting effector T cells and additional monocytes [24].

Sustaining this feedback loop also requires continued activity of the myeloid TLR4/HIF-1α axis, which favors accumulation of pro-inflammatory M1-like macrophages over immunoregulatory subsets. This pathway acts bidirectionally on adaptive immunity: It promotes infiltration and expansion of CD8^+^ cytotoxic and Th1 cells, worsening interstitial inflammation and tubular injury, while simultaneously suppressing regulatory T cell (Treg) and Th2 development, undermining the balance needed for transplant tolerance [25].

The CXCL10^+^LILRB2^+^ macrophage-centered chemokine network thus does more than sustain immune infiltration; it forges a reinforcing loop between innate and adaptive immunity that drives TCMR progression, making this network a potential therapeutic target.

### 3.4. Dysregulated repair and chronic fibrotic remodeling

When acute inflammation fails to resolve promptly, macrophage function pivots from inflammatory activation toward tissue remodeling, setting the stage for chronic fibrosis. In later stages of rejection, shifting microenvironmental cues push a subset of macrophages toward an anti-inflammatory but pro-fibrotic M2-like phenotype. Their persistent accumulation in the renal interstitium drives fibrosis through secretion of TGF-β1 and PDGF, which promotes epithelial-to-mesenchymal transition in tubular epithelial cells and activates fibroblasts, accelerating tubular atrophy and interstitial fibrosis and marking the shift from acute injury to chronic structural deterioration [31].

Beyond these paracrine effects, M2-like macrophages can themselves convert phenotype during chronic injury. The RNA m6A methyltransferase METTL3 is markedly upregulated in M2-like macrophages under chronic allograft damage, and its heightened activity has been linked to macrophage-to-myofibroblast transition (MMT), likely *via* activation of TGF-β1/Smad3 signaling. Once converted, these cells take on myofibroblast-like features and deposit extracellular matrix, producing type I collagen and α-smooth muscle actin (α-SMA), thereby contributing directly to interstitial matrix deposition and establishing themselves as a significant cellular source of interstitial collagen and renal fibrosis (Figure 2) [32].

**Figure 2.**
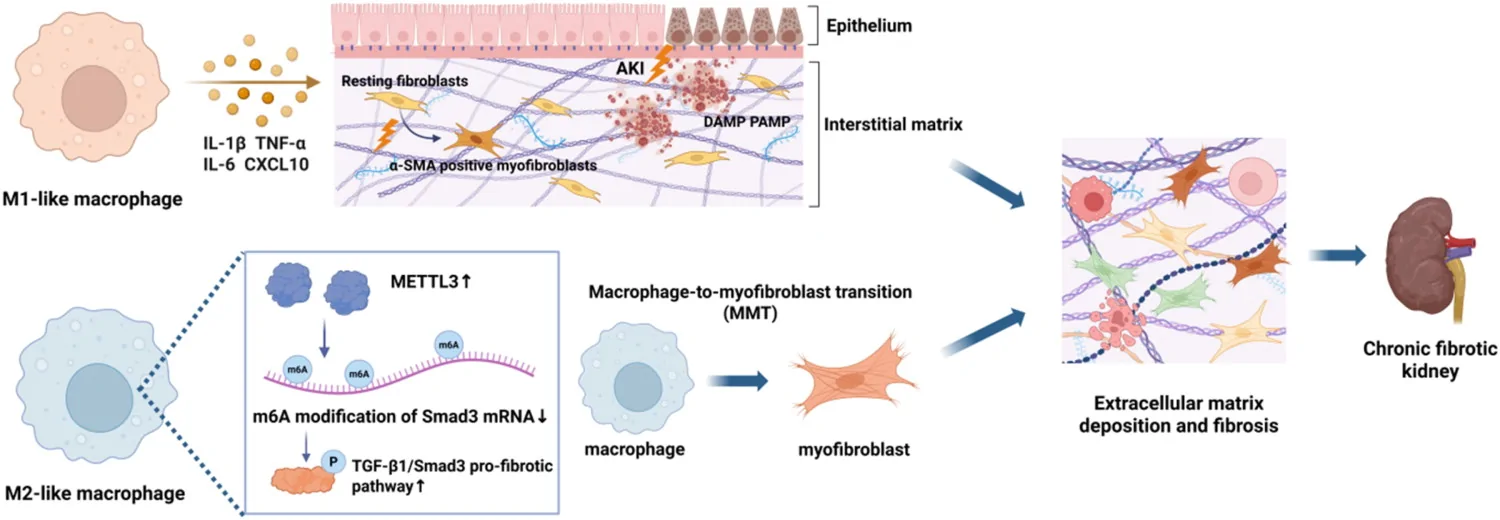
**Macrophage-Mediated Renal Injury and Fibrotic Remodeling in Renal Allograft TCMR.** M1-like macrophages amplify inflammatory renal injury, whereas M2-like macrophages promote fibrotic remodeling through METTL3-associated TGF-β1/Smad3 signaling and macrophage-to-myofibroblast transition (MMT), ultimately contributing to extracellular matrix deposition and chronic graft fibrosis.

Meanwhile, macrophages subjected to ischemic and rejection-associated injury sustain the expression of allograft inflammatory factor-1 (AIF-1), which continuously activates canonical Wnt/β-catenin signaling to lock in a pro-fibrotic phenotype and impairs the cells’ normal roles in clearing necrotic debris and supporting epithelial regeneration [33]. Together, METTL3/TGF-β1/Smad3-driven MMT and AIF-1/Wnt/β-catenin signaling promote maladaptive repair after rejection, culminating in irreversible interstitial fibrosis and tubular atrophy.

In the late phase of rejection, then, macrophages transition from inflammatory effectors into principal regulators of tissue remodeling, with sustained MMT and pro-fibrotic signaling together driving the progression from acute injury to chronic graft dysfunction.

Taken together, macrophages emerge not as passive downstream effectors but as principal hubs coordinating antigen presentation, parenchymal injury, immune amplification, and post-rejection fibrosis throughout TCMR. Given their dual capacity for both injury and repair, therapeutic strategies may need to move beyond indiscriminate macrophage depletion toward selective reprogramming of specific phenotypes or signaling pathways. This approach could suppress pathological rejection while preserving reparative function, offering a path beyond the current limits on long-term graft survival.

## 4. Application and engineering strategies of chimeric antigen receptor macrophages (CAR-Ms) in transplant immunology

Given their involvement at nearly every stage of TCMR, macrophages have increasingly emerged as the attractive targets for immunomodulatory intervention [22]. Current approaches, however, rely largely on conventional immunosuppressants or single-pathway inhibitors: Glucocorticoids broadly suppress macrophage activation *via* NF-κB inhibition, mycophenolate mofetil limits monocyte proliferation and differentiation, and sirolimus targets mTOR to restrain M1 polarization [34]. Because each acts on a single pathway, none achieves durable correction of the dysregulated intragraft immune microenvironment [35].

Chimeric antigen receptor macrophages (CAR-Ms) offer a potential cellular-engineering platform for more precise immune modulation in transplantation. Unlike CAR-T cells, whose efficacy depends chiefly on cytotoxic killing, CAR-Ms retain the native repertoire of macrophage functions, phagocytosis, antigen presentation, and immune regulation, along with an inherent adaptability to solid tissue microenvironments that may make them suitable, in principle, for complex immune-mediated conditions such as TCMR.

### 4.1. Basic structure, manufacturing, and delivery platforms of CAR-Ms

The CAR is a synthetic transmembrane receptor whose structural design governs antigen recognition efficiency, signaling strength, and the resulting macrophage polarization. Unlike endogenous TCRs, which recognize antigen in an MHC-restricted manner, CARs bypass MHC dependency altogether, using an antibody-derived extracellular single-chain variable fragment (scFv) to recognize surface antigens directly [36].

A conventional CAR comprises three domains: extracellular, transmembrane, and intracellular (ICD). The extracellular domain, built from the scFv and a flexible hinge, handles antigen recognition; the scFv itself joins the immunoglobulin heavy- and light-chain variable regions (VH/VL) *via* a peptide linker, preserving specificity and affinity while allowing the recognition domain to be swapped out to redirect the CAR toward new targets. The hinge, typically derived from IgG Fc or the extracellular segments of CD8α or CD28, links the scFv to the transmembrane domain and governs receptor flexibility, allowing engagement with epitopes at varying spatial positions on the target cell.

The transmembrane domain, commonly built from CD4, CD8α, or CD28, anchors the receptor in the macrophage membrane and relays signal from the extracellular domain inward, also shaping downstream signaling strength and receptor stability [37]. The intracellular domain then translates antigen engagement into macrophage effector function, phagocytic activity, polarization state, and anti-pathological output [ 37.]

Manufacturing CAR-Ms proceeds through CAR design, gene synthesis and cloning, vector construction, and genetic modification of macrophages: The synthetic CAR cassette is assembled and cloned into an expression vector, and then delivered into macrophages *via* viral or emerging non-viral platforms, yielding engineered cells that express functional CAR on their surface for antigen-specific recognition and activation.

Clinical translation, however, has been constrained by the biology of terminally differentiated macrophages, limited expansion, poor *in vivo* persistence, and inefficient genetic modification. Scalable engineering platforms based on granulocyte–monocyte progenitors (GMPs) address these limits: Engineered GMPs engraft within hematopoietic niches and continuously generate donor-derived mature macrophages and myeloid effector cells *in vivo*, sustaining long-term immune reconstitution across disease models and providing a technical foundation for durable CAR-M engineering and replenishment [38].

Delivery is equally consequential. CAR-Ms can be administered *via ex vivo* modification followed by adoptive transfer, or potentially generated *in situ* through targeted nanoparticle delivery. Lipid nanoparticles (LNPs) and hybrid membrane-based platforms show particular promise for improving engineering efficiency and tissue targeting: Ionizable or targeted LNPs carrying CAR mRNA enable transient, non-viral reprogramming while avoiding the risks of genomic integration [39], and hybrid nanocarriers combining plant-derived thylakoid membranes with liposomes may couple antioxidant and anti-inflammatory activity, scavenging local ROS while enabling efficient membrane fusion and targeted delivery, offering potential value in ischemia-reperfusion injury [40].

CAR-M therapy has already made substantial headway in oncology and inflammatory disease, typically by engineering enhanced antigen-specific phagocytosis and cytotoxicity through activation toward inflammatory M1-like states [41]. Renal transplantation, however, likely demands the opposite orientation: Rather than amplifying macrophage-mediated killing, the goal would be reprogramming macrophages away from M1-like inflammation and toward regulatory, tissue-protective phenotypes. This application remains essentially unexplored, and no CAR-M design tailored specifically to TCMR yet exists.

Given the stage-specific pathology of TCMR, we propose two complementary engineering strategies. The first, targeted-elimination CAR-Ms, would directly recognize and remove pathogenic effector immune cells to rapidly shut down acute inflammation. The second, tolerance-inducing resident CAR-Ms, would instead reshape the local immune microenvironment to foster durable tolerance. The following sections outline these two conceptual platforms and their potential optimization across three structural axes: the extracellular recognition domain, the transmembrane domain, and the intracellular signaling domain.

Although the present review focuses on TCMR, the broader involvement of macrophages in mixed cellular and humoral rejection also raises the possibility that CAR-M-based immunomodulation could eventually have cross-applicability in mixed rejection, potentially complementing therapies directed against antibody-mediated injury.

### 4.2. Design and precision elimination strategies of targeted-deletion CAR-Ms

The proposed goal for targeted-elimination CAR-Ms is straightforward in principle: selectively recognize and remove the pathogenic effector cells driving acute graft injury while sparing the surrounding immune microenvironment.

Because CD8^+^ effector T cells heavily infiltrate the graft during TCMR, CD8A/CD8B, the defining surface markers of cytotoxic T lymphocytes (CTLs) represent a potential extracellular target; an anti-CD8 scFv could in principle equip the CAR-M to recognize pathogenic CTLs directly [42]. A hinge derived from native CD8α would provide suitable flexibility for stable scFv–antigen engagement [36], while a CD86-derived transmembrane domain might improve CAR stability and compatibility with endogenous macrophage membrane trafficking.

On the intracellular side, pairing Fc receptor γ (FcRγ) with Megf10 might enhance target clearance: The ITAM motif within FcRγ would strengthen phagocytic signaling upon antigen engagement, while Megf10-linked efferocytosis pathways could promote efficient clearance with less collateral inflammatory mediator release [43]. Combining these two modules might thus improve pathogenic cell elimination while limiting local inflammatory amplification, though the specificity, efficacy, and safety of such constructs remain to be tested experimentally.

Importantly, however, CD8A/CD8B should be regarded as proof-of-concept recognition targets rather than rejection-specific therapeutic antigens. CD8 is broadly expressed by cytotoxic T-cell populations that mediate antiviral, antitumor, and other protective immune responses; consequently, unrestricted CD8-directed CAR-M activity could deplete protective T cells and compromise systemic immune surveillance. Greater selectivity would therefore require additional spatial, temporal, or molecular constraints. Graft-localized delivery could restrict CAR-M exposure to the transplanted kidney and reduce systemic contact with circulating protective CD8^+^ T-cell populations. Such localization might be achieved through direct graft-directed administration or delivery systems designed to preferentially accumulate within the renal allograft, thereby concentrating CAR-M activity at the site of rejection. Transient CAR expression using mRNA-based engineering could provide an additional temporal safeguard by limiting the duration of CD8-directed activity and allowing CAR expression to decline if off-target immune depletion occurs. Because mRNA-based expression is non-integrating and temporary, this approach may be particularly useful during acute rejection episodes in which short-term immune modulation is desired. Recognition of activation-associated or donor-reactive T-cell phenotypes may provide greater discrimination than CD8 alone.

Synthetic logic-gated recognition may offer an additional strategy to improve specificity by requiring the integration of multiple rejection-associated signals before full macrophage effector activity is initiated. For example, dual-target architecture could incorporate two extracellular recognition modules, with one recognizing CD8 and the other recognizing an additional rejection-associated surface marker. In an AND-gated design, engagement of either target alone would be insufficient to trigger full phagocytic signaling, whereas simultaneous recognition of both targets would activate the macrophage effector program. Such a dual-input strategy could, in principle, improve discrimination between pathogenic activated cytotoxic T cells and resting or protective CD8^+^ T-cell populations. Thus, CD8-directed CAR-Ms are presented here as an illustrative engineering framework rather than a clinically optimized targeting strategy, and their potential advantage over conventional systemic immunosuppression would depend on achieving substantially greater specificity for pathogenic alloreactive T cells.

### 4.3. Design and targeted immunomodulatory strategies of tolerance-inducing resident CAR-Ms

Tolerance-inducing resident CAR-Ms take a different approach: Rather than eliminating immune cells, they aim to reshape the local microenvironment toward durable graft acceptance. Conceptually, four TCMR-relevant processes offer engineering handles – curbing excessive antigen presentation, promoting anti-inflammatory polarization, disrupting recruitment circuits, and blocking fibrotic remodeling.

Acting on these processes would position such CAR-Ms as immune modulators rather than cytotoxic effectors, with the goal of restoring homeostasis while preserving macrophages’ native repair capacity. For extracellular recognition, tolerance-inducing CAR-Ms could in principle be directed toward graft-associated alloantigens or molecular cues enriched in the rejection microenvironment. For example, donor HLA could serve as a graft-associated recognition cue, whereas inflammatory or activation-associated molecules may provide additional context-dependent signals. However, donor HLA is broadly expressed by healthy graft parenchymal and endothelial cells and therefore lacks rejection specificity when used alone. Greater specificity could potentially be achieved by integrating multiple rejection-associated cues through synthetic logic-gated recognition. For instance, donor-HLA recognition could be combined with a second inflammatory or activation-associated signal enriched within the rejecting graft, such that full CAR-M activation would occur only when both signals are present.

More importantly, the purpose of this tolerance-inducing CAR-M design is fundamentally different from that of conventional pro-phagocytic CAR-M platforms. Extracellular recognition is not intended to trigger phagocytosis of the recognized graft cells. Instead, recognition would be functionally uncoupled from canonical phagocytic signaling pathways and used primarily to provide spatial and contextual control of CAR-M activation. Accordingly, the proposed design follows recognition–signaling–functional output logic: Extracellular cues determine where and under what inflammatory context the engineered macrophage is activated, the intracellular synthetic module determines how the recognition signal is interpreted, and the resulting functional output is directed toward immunoregulation and tissue protection rather than phagocytic elimination. The intracellular signaling module would therefore be redesigned to initiate anti-inflammatory, inhibitory, or tissue-protective programs rather than conventional phagocytic effector activity [44].This architecture is conceptually distinct from established CAR-M platforms that predominantly enhance antigen-dependent phagocytosis and inflammatory activity and therefore requires experimental validation. A macrophage-compatible transmembrane domain could be selected to support stable receptor expression while preserving the intended regulatory signaling architecture.

#### 4.3.1. Suppression of macrophage antigen-presenting capacity

Synthetic circuits modeled on IL-1R8 inhibitory signaling or TLR4/HIF-1α modulation represent one plausible engineering direction for curbing excessive alloimmune activation. Inhibiting TLR4/HIF-1α signaling could reduce MHC-II expression and blunt antigen-presenting activity, limiting downstream alloreactive T cell priming. At the same time, IL-1R8-like inhibitory circuits could similarly restrain macrophage-driven antigen presentation, preventing sustained pathogenic T cell activation and easing immune-mediated tubular injury [26].

#### 4.3.2. Induction of anti-inflammatory macrophage polarization

Modules that activate Smad2/3 signaling or block ERK1/2-FOS signaling might shift macrophages toward reparative states. Smad2/3 activation would mimic the protective effect of endogenous GDF15, suppressing M1 polarization in favor of anti-inflammatory programs; conversely, blocking ERK1/2-FOS could lower PHGDH expression, inhibit TLR4-mediated NLRP3 inflammasome activation, reduce caspase-1 cleavage, and limit release of IL-1β and IL-18, together curbing excessive M1 polarization [29].

#### 4.3.3. Disruption of macrophage–T cell recruitment circuits

Modules antagonizing LILRB2 signaling could interrupt the positive feedback loop between innate and adaptive immunity by blocking recipient CXCL10^+^ macrophages from recognizing donor HLA-I and driving effector T cell expansion. Modules that boost Zfp36 expression could further accelerate IL-27 p28 mRNA degradation, reducing CD8^+^ T cell activation, proliferation, and infiltration, jointly disrupting the macrophage–adaptive immunity feedback loop [27].

#### 4.3.4. Inhibition of macrophage-to-myofibroblast transition and chronic fibrosis

Intracellular domains targeting METTL3- and AIF-1-linked pro-fibrotic pathways might limit long-term fibrotic remodeling. Since macrophage METTL3 drives MMT *via* sustained TGF-β1/Smad3 activation, modules limiting Smad3 activity could suppress M2-to-myofibroblast conversion and reduce excessive matrix deposition; inhibiting the AIF-1-driven Wnt/β-catenin pathway could likewise promote reparative markers such as Arg1 and CD206 and enhance efferocytosis. Together, such strategies might help decouple inflammatory resolution from pathological fibrosis and support regenerative repair instead [32].

These four designs remain mechanism-guided concepts grounded in current understanding of macrophage biology in TCMR, and their feasibility, functional stability, and safety will require rigorous validation *in vitro* and *in vivo* (Figure 3).

**Figure 3.**
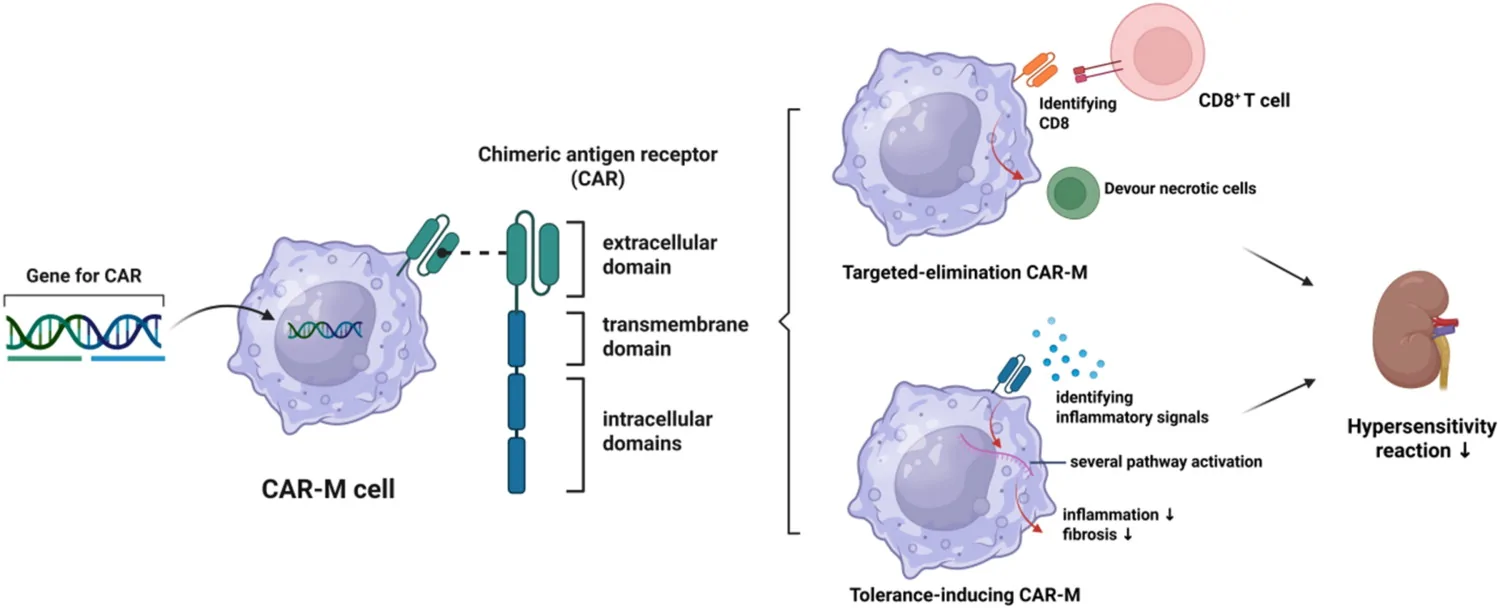
**Proposed CAR-Macrophage Engineering Strategies for the Treatment of Renal Allograft TCMR.** CAR-Ms are engineered with extracellular recognition, transmembrane, and intracellular signaling domains. Two proposed strategies include targeted-elimination CAR-Ms for removing pathogenic effector cells and tolerance-inducing CAR-Ms for suppressing inflammation and fibrosis and promoting graft tolerance.

### 4.4. Challenges and future perspectives of CAR-M translation

Despite their potential for precision immunomodulation in renal allograft TCMR, CAR-Ms face several hurdles before clinical translation is feasible. Target identification is the most fundamental: Unlike tumor antigens, which are often selectively enriched on malignant cells, TCMR-relevant targets are usually shared with physiological immune populations; CD8-directed strategies, for instance, might eliminate pathogenic CTLs while also compromising protective immune surveillance. Future platforms will likely need sharper target selection alongside context-dependent activation systems or logic-gated synthetic circuits to achieve adequate specificity without excessive off-target effects.

Macrophage plasticity presents an additional challenge. The same adaptability that lets engineered cells respond to complex graft microenvironments also invites functional drift after transplantation, as the cytokines, metabolic cues, and DAMPs pervading a rejecting graft can reshape CAR-M phenotype and undercut its intended function. Understanding how engineered macrophages behave once exposed to the transplant microenvironment, and developing ways to lock in stable function, will be a priority going forward.

A further translational challenge is host immune rejection of allogeneic CAR-M products, particularly if scalable off-the-shelf platforms are envisioned. Unlike autologous products, CAR-Ms generated from unrelated donor monocytes, hematopoietic progenitors, or pluripotent stem cell-derived sources may be recognized as non-self by the recipient immune system. Mismatched HLA molecules can trigger T-cell-mediated allorecognition and elimination of the infused cells, while humoral responses against donor alloantigens may further promote antibody-mediated clearance. NK cells may also contribute to rejection, particularly when HLA class I expression is reduced and inhibitory self-recognition signals are lost [45]. These adaptive and innate immune responses may limit CAR-M persistence and could become particularly relevant in settings requiring repeated administration.

Emerging hypoimmunogenic or ‘stealth’ engineering strategies may help mitigate these barriers. Reduction or deletion of HLA class I and class II expression, including approaches targeting B2M- or CIITA-dependent pathways, can decrease recognition by host T cells. However, extensive loss of HLA class I may increase susceptibility to NK-cell-mediated ‘missing-self’ recognition. Accordingly, multiplex engineering strategies have combined HLA reduction with expression of NK-inhibitory ligands such as HLA-E or HLA-G, while enhancement of antiphagocytic signals such as CD47 may provide an additional means of limiting host-mediated clearance. Stem cell- and progenitor-based platforms may be particularly amenable to such multigene engineering and standardized manufacturing [46]. Nevertheless, increasing immune evasion may introduce an important safety tradeoff, because engineered cells that resist host immune clearance may also become more difficult to control if unintended or dysregulated functions emerge. Future allogeneic CAR-M platforms will therefore need to balance reduced immunogenicity with preserved macrophage function, controllability, and long-term safety.

In addition to immunological compatibility, manufacturing, delivery, persistence, and safety remain major considerations for clinical translation.The optimal cellular source, mature macrophages, monocyte-derived cells, or hematopoietic progenitor-derived macrophages, still needs to be established, as do delivery systems capable of adequate graft localization and durable persistence. Continued advances in synthetic biology, biomaterials, and targeted delivery may help close these gaps and move CAR-M approaches from experimental concept toward clinically viable immunotherapy.

## 5. Conclusions and future perspectives

Through antigen presentation, inflammatory regulation, chemokine-mediated recruitment, phagocytosis, and tissue repair, macrophages participate across the full course of renal allograft TCMR. Their marked functional plasticity makes them both important contributors to graft injury and potential targets for cellular engineering. Targeted-elimination and tolerance-inducing resident CAR-Ms represent two conceptual strategies for harnessing that plasticity toward more precise control of the transplant immune microenvironment.

CAR-M-based intervention in kidney transplantation nonetheless remains an emerging concept requiring extensive experimental validation. Future work should focus on sharpening target specificity, stabilizing engineered macrophage function, improving delivery and persistence within graft tissue, and establishing long-term safety. Realizing this potential will depend on integrating advances across transplant immunology, synthetic biology, biomaterials, and cellular engineering.

CAR-M technology, in sum, provides a conceptual framework for complementing broad immunosuppression with more precise immune remodeling. Substantial challenges remain, but continued investigation into macrophage biology and engineering may ultimately support more durable graft acceptance and improved transplant outcomes.

## Data Availability

Data sharing is not applicable to this article as no new data were created or analyzed in this study. All data cited in this review are derived from publicly available published literature.
